# Ultrasound guidance aids in determining the success of equine temporomandibular joint arthrocentesis

**DOI:** 10.3389/fvets.2026.1812140

**Published:** 2026-05-20

**Authors:** Daniel E. Lopez, James L. Carmalt

**Affiliations:** 1 Department of Large Animal Clinical Sciences, Western College of Veterinary, University of Saskatchewan, Saskatoon, Canada; 2 Equi-CaRe, Fölziehausen, Germany

**Keywords:** arthrocentesis, equine, interventional ultrasonography, TMJ, ultrasound

## Abstract

**Background:**

The equine temporomandibular joint (TMJ) is divided into two non-communicating joint (discotemporal DTJ and discomandibular DMJ) compartments. Ultrasound has been extensively used as a diagnostic tool in the horse, but previous work has reported that ultrasound-guidance did not improve the success rate of TMJ arthrocentesis. However, this research focused only on the larger, easily accessed, DTJ compartment. However, most of the osseous pathology of the equine TMJ has been reported to be in the smaller DMJ.

**Objective:**

To determine whether using ultrasound-guidance would reduce the number of attempts to successfully perform arthrocentesis of the equine TMJ, specifically the DMJ.

**Study design:**

Experimental study using cadaver animals.

**Methods:**

The TMJ regions of seven thawed cadaver heads were clipped and cleansed. Arthrocentesis of the left DTJ and DMJ was attempted before repeating the attempts using ultrasound guidance. The number of attempts required to perform successful arthrocentesis of each joint compartment was documented before the second operator performed the same task. The same procedures were then performed on the opposite side of the head.

**Results:**

As operators gained experience, their success rate climbed. Statistically, none of the other documented outcome variables significantly affected the number of attempts to obtain successful arthrocentesis. Despite that, ultrasound-guidance allowed immediate unequivocal evidence of successful arthrocentesis into the targeted joint compartment.

**Main limitations:**

Low number of normal cadaver horse heads and a lack of randomization of attempt techniques.

**Conclusions:**

This study shows that ultrasound-guidance did not reduce the number of attempts required for successful DTJ, or DMJ arthrocentesis. However, it did allow for the immediate unequivocal evidence of successful arthrocentesis into the targeted joint compartment.

## 1 Introduction

The equine temporomandibular joint (TMJ) is comprised of two independent joint compartments. The larger (discotemporal joint - DTJ), is bounded dorsally by the mandibular fossa of the zygomatic process of the temporal bone. The smaller (discomandibular joint—DMJ) is situated above the condylar process of the mandible and both are completely separated from each other by a bi-concave, fibrocartilagenous, intra-articular disc (1). Initial publications focused almost exclusively on the diagnosis and treatment of septic joints (2–5). More recently intra-articular pathology (cysts) and attendant osteoarthritis have been reported as causes of poor performance in sport horses (6, 7). As awareness of equine TMJ disease increases more clinicians are performing arthrocentesis when pursuing further diagnostic tests or treating this joint (8).

In previous publications successful TMJ arthrocentesis has been documented using computed tomography (1, 9) or radiography (10). Weller et al. (1999) described three acoustic windows (rostrolateral, lateral, and caudolateral) for evaluation of the equine TMJ (11), and their use has been reported widely in clinical case reports, including as an additional diagnostic and follow-up tool in cases of septic arthritis (12) and for TMJ arthroscopic portal placement under ultrasound guidance (13); however, ultrasound has not been commonly used for guidance of arthrocentesis.

Norvall et al. (2020) reported that ultrasound guidance did not decrease the number of attempts to successfully inject the equine TMJ (9). While the specific joint compartment injected was not reported in that paper, the authors used the Rosenstein technique (2001) which utilizes a lateral approach to the caudal pouch of the DTJ (14). Given that the equine TMJ is comprised of two separate synovial compartments (1) and that 80% of non-septic equine TMJ pathology occurs in the DMJ (15), ultrasound-guidance has been postulated as a useful method of accessing this smaller, relatively inaccessible, joint compartment (10), as has been reported in the human literature (16).

There are two reported approaches to arthrocentesis of the equine DMJ. A caudodorsal to rostroventral approach (17) to the caudal recess of the joint compartment through the DTJ, and a direct lateromedial approach (10). Both require experience, the ability to palpate the joint margins, and an appreciation of anatomy.

The objective of this study was to determine whether using ultrasound-guidance would alter the number of attempts to successfully achieve TMJ arthrocentesis (DTJ and DMJ). The allied hypothesis was that ultrasound guidance would reduce the number of attempts to achieve successful TMJ arthrocentesis (DTJ and DMJ).

## 2 Materials and methods

The study population comprised of seven cadaver heads from a selection which had been euthanized for reasons unrelated to the study and kept, with owner permission, for instructional purposes. The estimated age ranged from 1 to 25 years old (14 years, SD +/−7.7), based on dental examination. Heads were placed on the edge of a steel table on their mandibles. Using a full-mouth speculum (Stubbs Equine Innovations, Johnson City, TX) the mandible of each head was opened maximally prior to intervention to allow unimpeded movement of the jaw which allowed for delineation of the articular margins and intra-articular disc. Two investigators (JLC and DEL) experienced in performing equine arthrocentesis performed all arthrocentesis procedures; however, DEL had not injected equine TMJs prior to performing the study.

The TMJ regions of the cadaver heads were clipped and cleansed before having ultrasound coupling gel applied. The mandibular condyle and intra-articular disc of the left and right TMJs were identified by palpation and a 22-gauge, 1.5-inch needle was used to attempt arthrocentesis of the DTJ of the left TMJ using a lateral approach (14). An attempt was determined to be insertion of the needle through the skin with minimal re-direction of the tip. If more than two re-directions were required, then the needle was removed and a second attempt was performed. If more than three attempts (6 re-directions) were performed without successful joint entry the arthrocentesis was deemed a failure.

**Figure 1:**
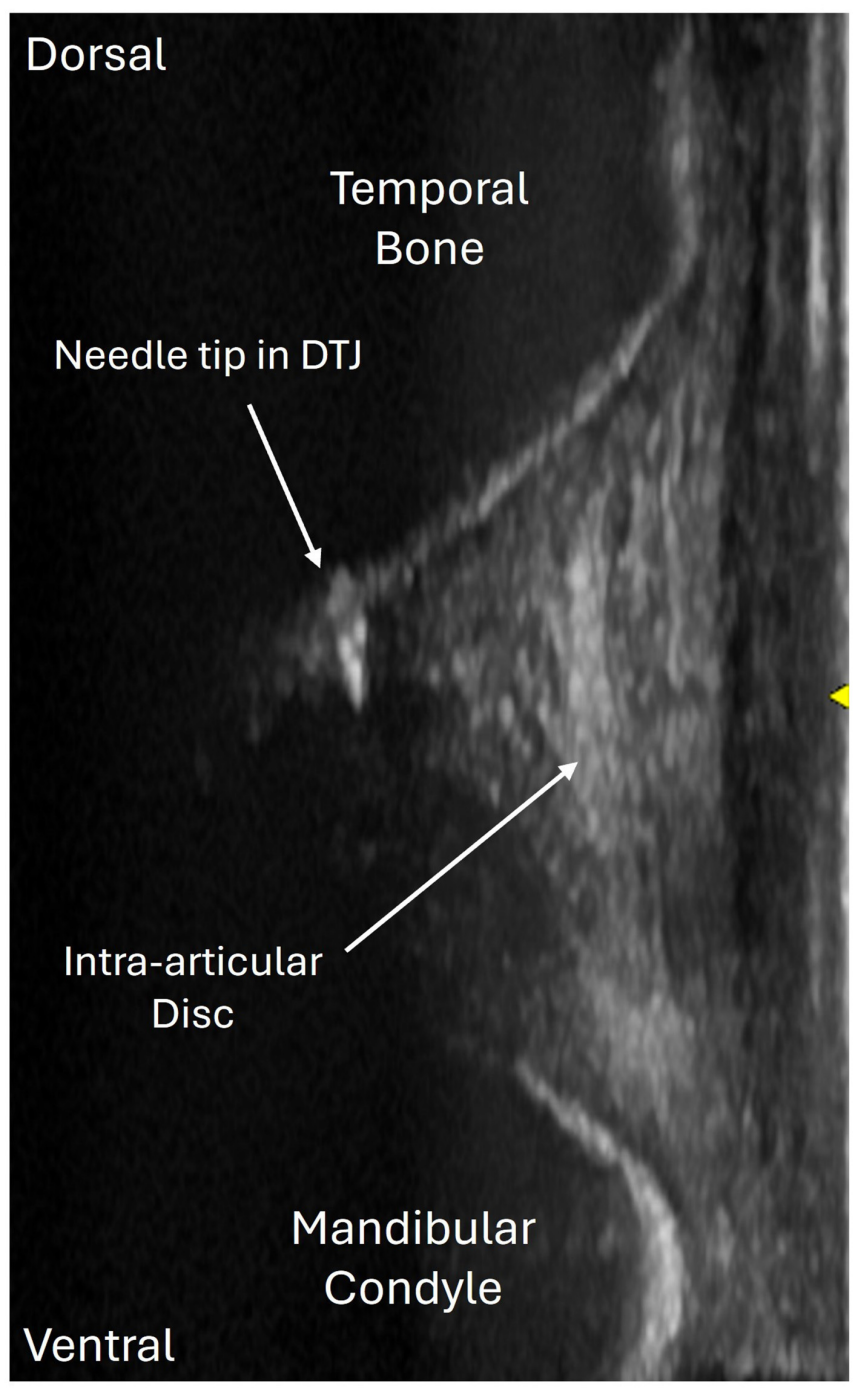
An ultrasonographic image of an intra-articular needle within the discotemporal joint (DTJ) compartment of the left temporomandibular joint.

3 ml of colored (methylene blue) liquid injectate was used. A lack of resistance to fluid flow and ease of retrieval of the injectate was used as an indication of successful entry into the joint which was ultimately confirmed using ultrasound (B-mode, 13 MHz, (12L-RS wideband linear-array transducer 6–13 MHz, GE Healthcare, Chicago, IL, USA) with gain, focal zone and depth optimized for each cadaver). The same investigator then attempted arthrocentesis of the DMJ (10) assessing success as above. Then both joints were approached again using ultrasound guidance. The ultrasound probe was positioned in the caudolateral window as previously described by Weller et al. (1999), with the transducer oriented vertically, and no standoff pad was used (11). This orientation allowed visualization of the lateral aspect of the mandibular fossa of the temporal bone dorsally, the articular disc centrally, and the mandibular condyle ventrally. The target joint space was centered within the imaging field using the on-screen targeting marker. The needle was inserted using the central needle guide marker on the transducer as a reference point, just rostral to the transducer, and advanced perpendicular to the transducer in a lateromedial direction (Figure 1).

The order in which the investigators performed the arthrocentesis was randomized, but that of the injection technique (blind vs. ultrasound-guided) was not. The number of attempts to perform successful arthrocentesis of each joint compartment with, and without, ultrasound guidance were documented. The procedure was then repeated by the second investigator before moving on to the right side of the head.

Data were entered into a spreadsheet using Microsoft Excel (Microsoft Corporation, Redmond, WA, USA) and moved into a windows-based statistical program (Statistix 10, Analytical Software, Tallahassee, FL) for analysis. Wilcoxon rank sum tests for non-parametric data were used to examine the effects of the categorical variables (experience of operator, joint pouch, ultrasound guidance) on the outcome variable (number of attempts to achieve successful arthrocentesis). Statistical significance was set at *P* < 0.05. This simple series of statistical tests were chosen due to the limitation of the data. Multivariable regression analysis, such as ordinal logistic, or Poisson regression, were inappropriate as their assumptions could not be met by the data.

## 3 Results

There were seven horses with 14 TMJs and 28 joint compartments. Each was attempted twice (blind and ultrasound-guided) for a total of 56 measurements per operator. A total of 112 attempts were thus recorded.

Eleven attempts were deemed failures. Of these, nine took more than three independent needle placements which would be considered unacceptable to the authors in a clinical situation. Seven of the nine were when the DMJs were the targeted joint. In the remaining 2 cases a joint cavity was entered, but it was not the cavity for which the operator was aiming and so these were also considered failed attempts.

Overall, the number of attempts dropped significantly with experience. The first 4 horses had a mean number of 1.44 needle placements (attempts) per success (+/–SD 0.76; median 1.0, range 1–3) whereas the last 3 required 1.1 (+/–SD 0.3; median 1.0, range 1–2, Z statistic 2.52, *p* = 0.01).

None of the measured outcome variables reached mathematical significance. The investigator with more experience in performing arthrocentesis of the TMJs required no fewer (mean +/–SD: 1.21+/–0.56; *n* = 50) attempts per joint than the less experienced investigator (1.22+/–0.56; *n* = 51; Z statistic = 0.18: *p* = 0.85). The DTJ required fewer attempts (1.19 +/–0.52; *n* = 49) than the DMJ (1.39+/–0.7; *n* = 52, Z = 1.64: *p* = 0.1). Ultrasound guidance resulted in slightly more attempts (1.32+/–0.65; *n* = 50) compared to those attempted without ultrasound (1.25+/–0.59; *n* = 51; Z = 0.54: *p* = 0.6). The median of all tests was 1.0 with a range of 1–3.

## 4 Discussion

There was no significant difference in the number of attempts to achieve successful arthrocentesis of either joint compartment by operator experience, or ultrasound guidance. As the study progressed the number of attempts required to obtain a successful arthrocentesis dropped, independent of the use of ultrasound. These results confirm those of Norvall et al. (2020) and lead to the rejection of the hypothesis (14).

The DTJ compartment of the TMJ, which was presumably approached by Norvall et al. (2020; 9), has easily palpable external landmarks (14) whereas the DMJ compartment does not (10, 17). In the current study the lateral approach (10) to the DMJ was used as it does not require perforating the intra-articular disc when driving the needle from dorsal to ventral which occurs in the original approach. Despite the subtle external landmarks used in the lateral approach neither investigator had significant problems with arthrocentesis, nor gained any benefit from ultrasound-guidance which was unexpected. Randomization is a statistical method central to clinical trial design and is used in an attempt to reduce allocation bias. In this case, the intervention order. In the current study, the order in which the investigators performed the arthrocentesis was randomized to attempt to reduce any bias associated with watching the other person finding their landmarks for joint injections. However, the order in which the injection technique (blind vs. ultrasound-guided) was performed, was not. This was not an oversight. Placement of the needle to perform blind (non-assisted) arthrocentesis is a skill based on knowledge of anatomy and palpation. The investigators were concerned that if the correct position for needle placement had been previously identified by ultrasound evaluation prior to their non-assisted attempt, then unfair advantage would have been gained and a bias toward success of the blind technique gained. This is not true of subsequently using ultrasound-guidance. The investigators would find the most suitable place for arthrocentesis based on the findings from the diagnostic imaging- assuming their understanding of ultrasound anatomy was sound. Ultimately, the study did not find a significant difference between the two techniques, however, it would have been more statistically robust if randomization had been applied across every facet of the interventions.

Both investigators in our study were experienced at performing equine arthrocentesis, with or without imaging-guidance. Even though the first author had never injected the TMJ compartments, and the senior author had considerable experience with the joint, the overall difference was not statistically significant. This suggests that if the anatomy is understood, palpation skills are transferable to other joints. It is highly likely that ultrasound-guidance might have been useful especially for the DMJ or a diseased joint if a person did not have experience with arthrocentesis; however, that facet of investigation was not the purpose of the current study. Both joint compartments of the equine TMJ have rostral and caudal outpouchings available for arthrocentesis. The caudal outpouchings are the ones typically used, and both could be imaged using the previously described caudolateral acoustic window (11). The current study found that this window was the most appropriate for confirming correct joint entry using the blind approach and for ultrasound-guided arthrocentesis when the needle was placed immediately rostral to the probe. In comparison, lateral or rostrolateral approaches provided a narrower ultrasound window and more limited space for needle positioning due to the small size of the joint and the restricted working area (data not shown). The use of an ultrasound standoff pad was initially attempted, as it was believed to provide a better image, but it was later discontinued, as using the linear transducer without a standoff pad provided good image quality in clipped and previously prepared ultrasound areas. The ease of confirmation, especially when the wrong compartment is inadvertently injected (as was the case in the current study), could be very useful. Given that most of the osseous pathology has been reported to occur in the DMJ (15), confirmation of correct joint entry, rather than relying simply on ease of injection and retrieval of injectate, could be important when approaching this joint. Indeed, two of our failures came in exactly this manner. We were about to categorize an attempt at DMJ arthrocentesis as a success due to the aforementioned criteria, only to find upon ultrasound assessment that the needle was in the DTJ. The use of a smaller linear transducer instead of a larger one may provide more working space in smaller horses facilitate probe positioning and needle alignment; but this was not evaluated in the present study.

Weaknesses of the current study are that it only has low numbers (*n* = 7) of non-diseased cadaver material and the number of needle redirections with each attempt were not documented. Any study is improved with a larger dataset which improves the likelihood of detecting a treatment effect. In the current study the investigators reached a threshold limit (learning) whereby they were achieving arthrocentesis with the first attempt with, or without, ultrasound-guidance. A *post-hoc* power calculation performed on the data obtained (confidence level of 95%, expected standard deviation of 0.62 and an accepted absolute error of 0.07) suggested that the number of attempts recorded between ultrasound-guided and non-guided arthrocentesis were so comparable that 302 cadavers would have been needed to be confident in our lack of statistical significance. Adding further numbers would lend more credibility to the results, especially if the cadaveric material was varied in its disease state, however the authors only had a sample of normal cadaveric material upon which to base their study. While limiting, from an experimental standpoint, this cadaver material more accurately mirrors the anatomy of our live patients who present for the investigation of subtle behavior, or masticatory, problems. Those animals, if they have early osteoarthritis, do not tend to have overt lateral osteophytosis or joint swelling. Obviously, if there are alterations in the contour of the joint (soft tissue or bony origin), ultrasound-guidance may be of benefit, but that was not assessed in the current study.

As stated, the number of needle redirections was not documented, which was regrettable. However, the investigators limited each attempt to two re-directions as they do in clinical situations. This reduces soft tissue trauma, prevents “fishing for the joint” and unnecessary discomfort to the patients.

Ultrasound-guidance has been used to perform arthrocentesis in human TMJ disease with varied success. Antony et al. (2019) reported a first time intra-articular needle placement success of 100% with ultrasound-guidance but only 60% without, albeit in the large upper (human) joint space (18). This contrasts with a report by Sivri et al. (2016) who also used the upper joint space and found that ultrasound-guided arthrocentesis was not more successful than the non-guided approach and took significantly longer (19). Our small study complimented that of Norvall et al. (2020) showing that ultrasound-guidance did not reduce the number of needle placements for successful arthrocentesis beyond the improvement gained with experience in the study (9). However, our experiences mirror those of Torres-Gaya et al. (2021) in their human study in which they report that ultrasound-guidance provided “image verification of having punctured the joint space…..distention of the space after infiltrating a small amount of fluid…and observing the corresponding slow-moving echoes in greyscale” (20). Further, they state that “knowing the exact depth of joint space and needle tip provides precision, safety and confidence…”. In our hands, ultrasound -guidance allowed immediate, categorical, determination of specific joint entry (DMJ vs. DTJ) as the injectate could be seen swirling within the joint, the intra-articular disc would move as the joint pressure increased, and the joint capsule would distend toward the ultrasound probe. This knowledge and confidence was a tangible benefit of ultrasound-guidance not captured in the purely statistical assessment of the current study. As such, when attempting to enter the smaller DMJ it may be worth using ultrasound-guidance, if only to confirm joint space entry.

## 5 Conclusion

This study showed that ultrasound-guidance did not reduce the number of attempts to successfully perform arthrocentesis of the TMJ joint compartments. If failure occurred, it was more likely to be with entry into the DMJ. Despite that, and similar to the findings in human studies, ultrasound-guidance using a caudal window was simple to perform and gave immediate peace of mind when it came to determining successful joint entry into the compartment that the operator was intending to target. Without it, an erroneous assumption of success could be made as injectate could be aspirated, but from the wrong joint compartment.

## Data Availability

The raw data supporting the conclusions of this article will be made available by the authors, without undue reservation.
